# Author Correction: Targeting de novo lipogenesis and the Lands cycle induces ferroptosis in KRAS-mutant lung cancer

**DOI:** 10.1038/s41467-022-32459-x

**Published:** 2022-08-08

**Authors:** Caterina Bartolacci, Cristina Andreani, Gonçalo Vale, Stefano Berto, Margherita Melegari, Anna Colleen Crouch, Dodge L. Baluya, George Kemble, Kurt Hodges, Jacqueline Starrett, Katerina Politi, Sandra L. Starnes, Daniele Lorenzini, Maria Gabriela Raso, Luisa M. Solis Soto, Carmen Behrens, Humam Kadara, Boning Gao, Ignacio I. Wistuba, John D. Minna, Jeffrey G. McDonald, Pier Paolo Scaglioni

**Affiliations:** 1 Department of Internal Medicine, University of Cincinnati College of Medicine, Cincinnati, OH 45219 USA; 2 Center for Human Nutrition, The University of Texas Southwestern Medical Center, Dallas, TX 75390 USA; 3 Department of Neuroscience, The University of Texas Southwestern Medical Center, Dallas, TX 75390 USA; 4 Department of Interventional Radiology, The University of Texas MD Anderson Cancer Center, Houston, TX USA; 5 Tissue Imaging and Proteomics Laboratory, Washington State University, Pullman, WA 99164 USA; 6 Sagimet Biosciences, San Mateo, CA 94402 USA; 7 Department of Pathology, University of Cincinnati College of Medicine, Cincinnati, OH 45219 USA; 8 Yale Cancer Center, Yale School of Medicine, New Haven, CT USA; 9 Department of Surgery, Division of Thoracic Surgery, University of Cincinnati College of Medicine, Cincinnati, OH 45219 USA; 10 Department of Pathology, Fondazione IRCCS Istituto Nazionale dei Tumori di Milano, via Venezian 1, 20133 Milan, Italy; 11 Department of Translational Molecular Pathology, The University of Texas MD Anderson Cancer Center, Houston, TX USA; 12 Department of Thoracic H&N Medical Oncology, The University of Texas MD Anderson Cancer Center, Houston, TX USA; 13 Hamon Center for Therapeutic Oncology Research, The University of Texas Southwestern Medical Center, Dallas, TX 75390 USA

**Keywords:** Non-small-cell lung cancer, Cancer metabolism, Targeted therapies

Correction to: *Nature Communications* 10.1038/s41467-022-31963-4, published online 26 July 2022

In the Acknowledgements section of this article the grant number relating to UT Southwestern Medical Center given for Pier Paolo Scaglioni was incorrectly given as CPRIT RP1606552 and should have been CPRIT RP160652. The original article has been corrected.

