# Supplementary material for: Targeting de novo lipogenesis and the Lands cycle induces ferroptosis in KRAS-mutant lung cancer
Source: Nat Commun. 2022 Jul 26;13:4327. doi: 10.1038/s41467-022-31963-4 (PMC9325712; doi:10.1038/s41467-022-31963-4)
Supplement: Supplementary file 1 — Supplementary Information [file 41467_2022_31963_MOESM1_ESM.pdf]

Supplementary information

Targeting *de novo* lipogenesis and the Lands cycle induces ferroptosis in KRAS-mutant lung cancer

Caterina Bartolacci[1,#], Cristina Andreani[1,#], Gonçalo Vias Do Vale[2], Stefano Berto[3], Margherita Melegari[1], Anna C. Crouch[4], Dodge L. Baluya[5], George Kemble[6], Kurt Hodges[7], Jacqueline Starrett[8], Katerina Politi[8], Sandra L. Starnes[9] ,Daniele Lorenzini[10], Maria Gabriela Raso[11], Luisa Solis Soto[11], Carmen Behrens[12], Humam Kadara[11], Boning Gao[13], Ignacio I. Wistuba[11], John D. Minna[13], Jeffrey McDonald[2], and Pier Paolo Scaglioni[1,*]

Affiliations:

[1] Department of Internal Medicine, University of Cincinnati College of Medicine, Cincinnati, OH 45219, USA.

[2] McDermott Center for Human Growth and Development, The University of Texas Southwestern Medical Center, Dallas, TX 75390, USA.

[3] Department of Neuroscience, The University of Texas Southwestern Medical Center, Dallas, TX 75390, USA

[4] Department of Interventional Radiology, The University of Texas MD Anderson Cancer Center

[5] Tissue Imaging and Proteomics Laboratory, Washington State University, Pullman, WA 99164.

[6] Sagimet Biosciences, San Mateo, CA 94402.

[7] Department of Pathology, University of Cincinnati College of Medicine, Cincinnati, OH 45219, USA.

[8] Yale Cancer Center, Yale School of Medicine, New Haven, Connecticut.

[9] Department of Surgery, Division of Thoracic Surgery, University of Cincinnati College of Medicine, Cincinnati, OH 45219, USA

[10] Department of Pathology, Fondazione IRCCS Istituto Nazionale dei Tumori di Milano, via Venezian 1, 20133 Milan, Italy

[11] Department of Translational Molecular Pathology,

[12] Department of Thoracic H&N Med Oncology, The University of Texas MD Anderson Cancer Center.

[13] Hamon Center for Therapeutic Oncology Research, The University of Texas Southwestern Medical Center, Dallas, TX 75390, USA.

[#] These authors contributed equally to this manuscript.

* Corresponding author: Pier Paolo Scaglioni, The Vontz Center for Molecular Studies, 3125 Eden Avenue, Rm. 3118, Cincinnati, OH 45219-2293. Email: Scaglipr@ucmail.uc.eduPhone: 513-558-2115; FAX: 513-558-2125.

Running title: Mutant KRAS lung cancer depends on FASN and the Lands cycle

Conflict of interest statement: The authors declare no potential conflicts of interest.

Content

Supplementary Tables 1 and 2

Supplementary Figures 1-11 and Supplementary Figure legends

Other Supplementary Materials for this manuscript include the following:

Supplementary Data 1 to 9 as Excel files

Supplementary Videos 1-3

Supplementary Table 1. List and characteristics of the primary human LC specimens.

Biobank ID	KM	Diagnosis	Morphology	Age at Collection	Gender	Race
L140	Yes	Lung Cancer	Adenocarcinoma	81	Female	White
L550	Yes	Lung Cancer	Adenocarcinoma	60	Female	White
THO744	Yes	Lung Cancer	Adenocarcinoma	60	Female	White
L569	No	Lung Cancer	Adenocarcinoma	70	Female	White
L584	No	Lung Cancer	Adenocarcinoma	79	Male	White
THO703	No	Lung Cancer Mets	Adenocarcinoma	67	Female	Asian

Supplementary Table 2. List and characteristics of the LC PDXs. NSCLC, non-small cell lung cancer; AD, adenocarcinoma

ID	Tumor type	KRAS status	EGFR status
HCC4256-PDX-F0	PDX-NSCLC	G12C	WT
LTL-618T	PDX-NSCLC	G12C	WT
HCC4058-PDX-F1	PDX-NSCLC	G12D	WT
HCC4059-PDX-F1	PDX-AD	G12V	WT
LTL-656T	PDX-AD	G12V	WT
HCC4271-PDX-F0	PDX-NSCLC	WT	WT
HCC4285-PDX-F0	PDX-AD	WT	WT
HCC4300-PDX-F0	PDX-NSCLC	WT	WT
HCC4307-PDX-F0	PDX-AD	WT	WT
HCC4190-PDX-F0	PDX-AD	WT	L858R
LTL-347T	PDX-AD	WT	L858R

Supplementary Figure 1

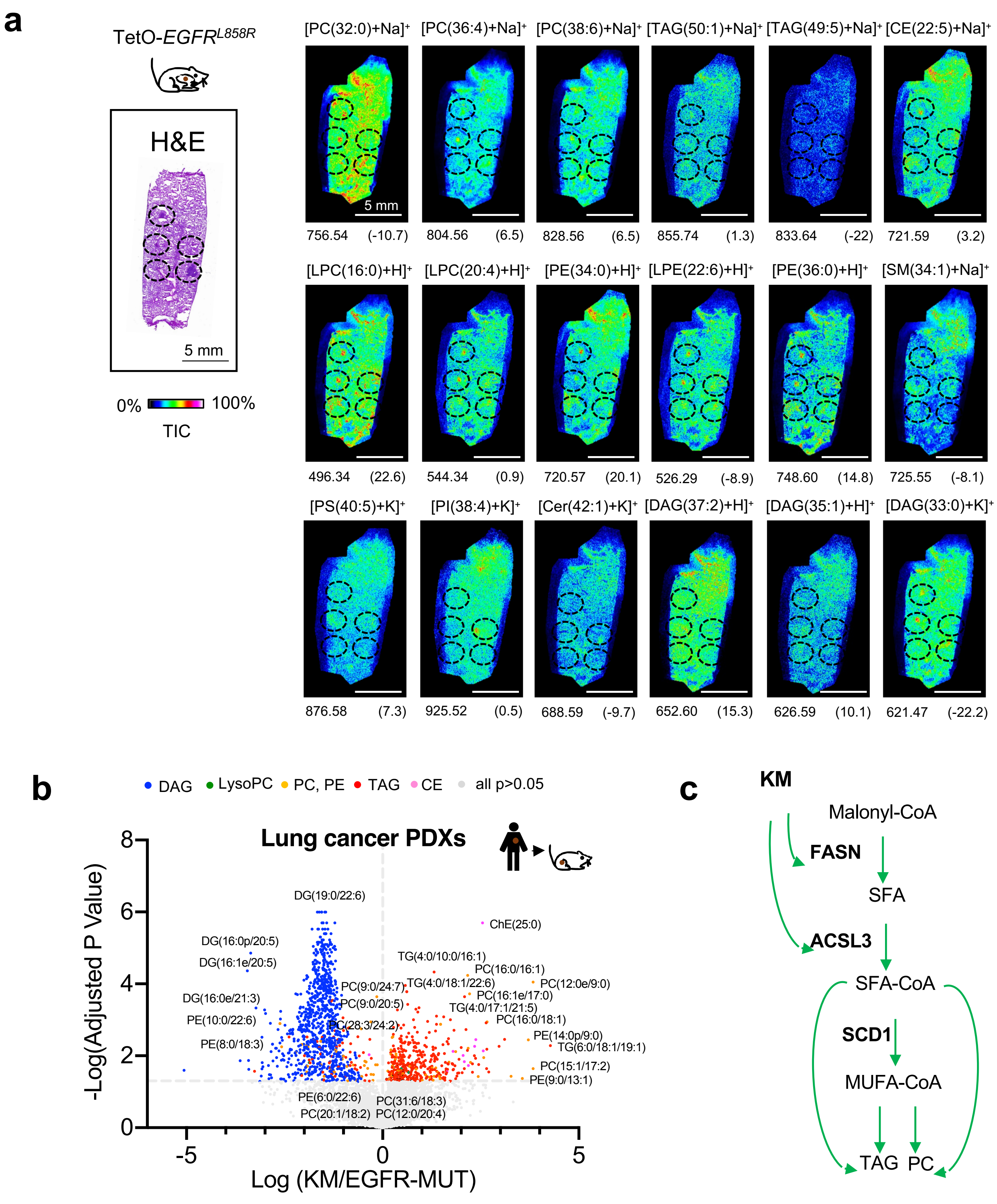

Supplementary Figure 1. Mutant EGFR LC and KMLC have distinct lipidomic profiles. (a) H&E staining and MALDI imaging pictures of TetO-*EGFR*^{L858R} lungs (n=2 biologically independent experiments). Rainbow scale represents % ion intensity normalized against the total ion count (TIC). Circles indicate tumor areas. Observed m/z and mass error (ppm) values are indicated for each lipid species. Refer to Supplementary Data 1 and 4 for the complete tentative MALDI lipid annotation and relative quantification **(b)** HPLC-MS/MS analysis of EGFR-MUT (n=2) and KM (n=5) lung cancer patient-derived xenografts (PDXs). P values and difference (EGFR-MUT *versus* KM) were calculated using multiple t tests ($p<0.05$). **(c)** Proposed mechanisms of KM-dependent regulation of lipid synthesis in lung cancer. KM activates *FASN* and *ACSL3* which synthesize saturated fatty acids (SFA) and SFA-CoAs respectively. SFA-CoAs are either desaturated by SCD1 to monounsaturated FA (MUFA-CoAs) or directly used for the synthesis of TAG and PC.

Supplementary Figure 2

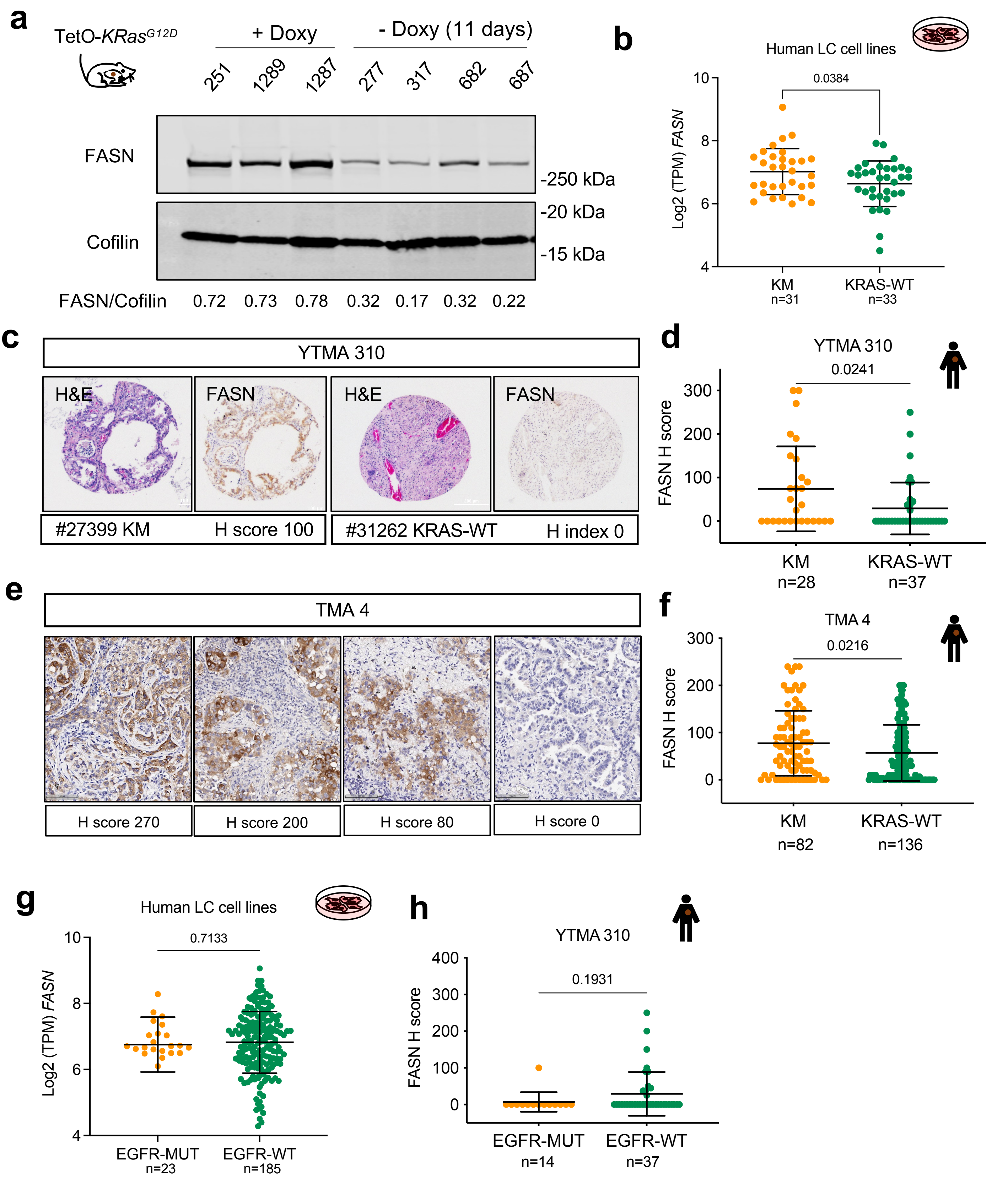

Supplementary Figure 2. FASN is upregulated in KMLC. (a) Immunoblot analysis of FASN in micro-dissected lung tumors of CC10rtTA; TetO-*Kras*^{G12D} mice +/-doxy for the indicated number of days (n=2 independent experiments). **(b)** FASN expression in human non-small cell lung cancer (NSCLC) cell lines from the cancer cell line encyclopedia (CCLE) of the indicated genotype. TPM, transcript per million. n=number of cell lines. **(c, d)** Representative images of FASN immunostaining in LC specimens of YTMA 310 and its H-score quantification. n=number of cores. **(e, f)** Representative images of FASN immunostaining in LC specimens of TMA 4 and its H-score quantification. n=number of cores. **(g)** *FASN* expression in human cell lung cancer cell lines from the cancer cell line encyclopedia (CCLE) of the indicated genotype. Data are expressed as mean ± SD. **(h)** FASN H-score quantification of EGFR-MUT and EGFR-WT cores in YTMA 310 (n=number of cores). In (b), (d), (f), (g), (h) two-tailed unpaired Student's t test.

Supplementary Figure 3

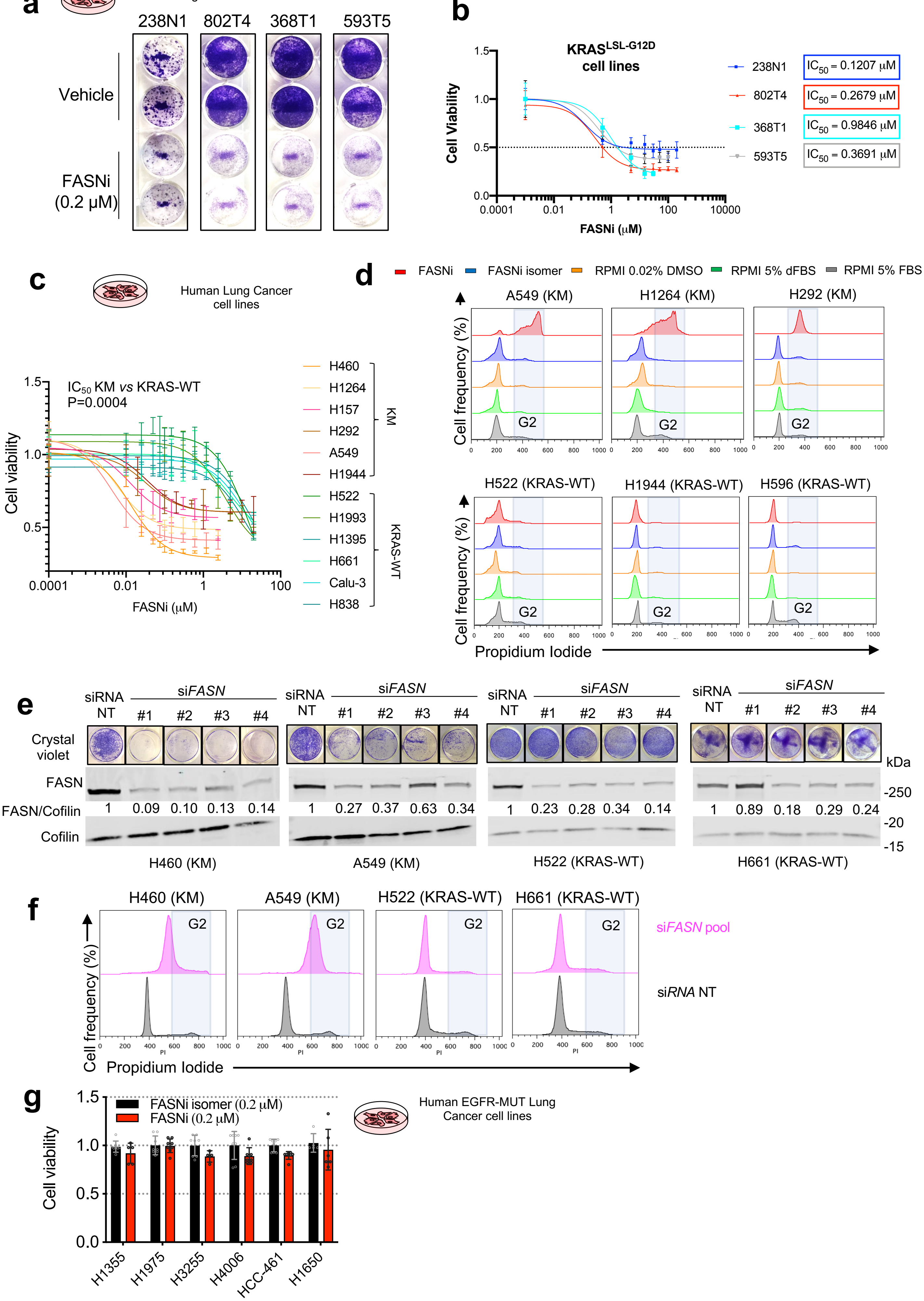

Supplementary Figure 3. FASN is a selective vulnerability of KMLC. (**a**) Crystal violet and (**b**) MTT viability assays of the indicated murine LSL-*Kras*^{G12D} cell lines treated as indicated. IC_{50}, inhibitory concentration 50 (n=4 biologically independent cell lines). (**c**) MTT viability assay on the indicated human KM and KRAS-WT LC cell lines treated as indicated (n=6 biologically independent cell lines each genotype). IC_{50} values were determined using non-linear regression and P value indicates two-tailed unpaired Student's t test of IC_{50} values KM *versus* KRAS-WT.
(**d**) Cell cycle analysis on the indicated human LC cell lines treated as indicated. RPMI 5% FBS = regular medium; RPMI 5% dFBS= medium with dialyzed FBS; RPMI 0.02% DMSO = vehicle. Cell populations indicate singlets in FL2-W/FL2-A gate. Refer to Supplementary Figure 11 for a representative gating strategy. (**e**) Crystal violet assay of the indicated cells transfected with the indicated siRNAs and correspondent immunoblot of FASN. NT, non-targeting. (**f**) Cell cycle analysis of KM and KRAS-WT LC cells transfected with the indicated siRNAs. siRNA FASN pool = siFASN #1-4. Cell populations indicate singlets in the FL2-W/FL2-A gate. Refer to Supplementary Figure 11 for a representative gating strategy. **(g)** Cell viability assay in EGFR-MUT LC cell lines treated as indicated for 7 days (n=6 biologically independent cell lines each genotype). In b, c, g data are represented as mean ± SD.

Supplementary Figure 4

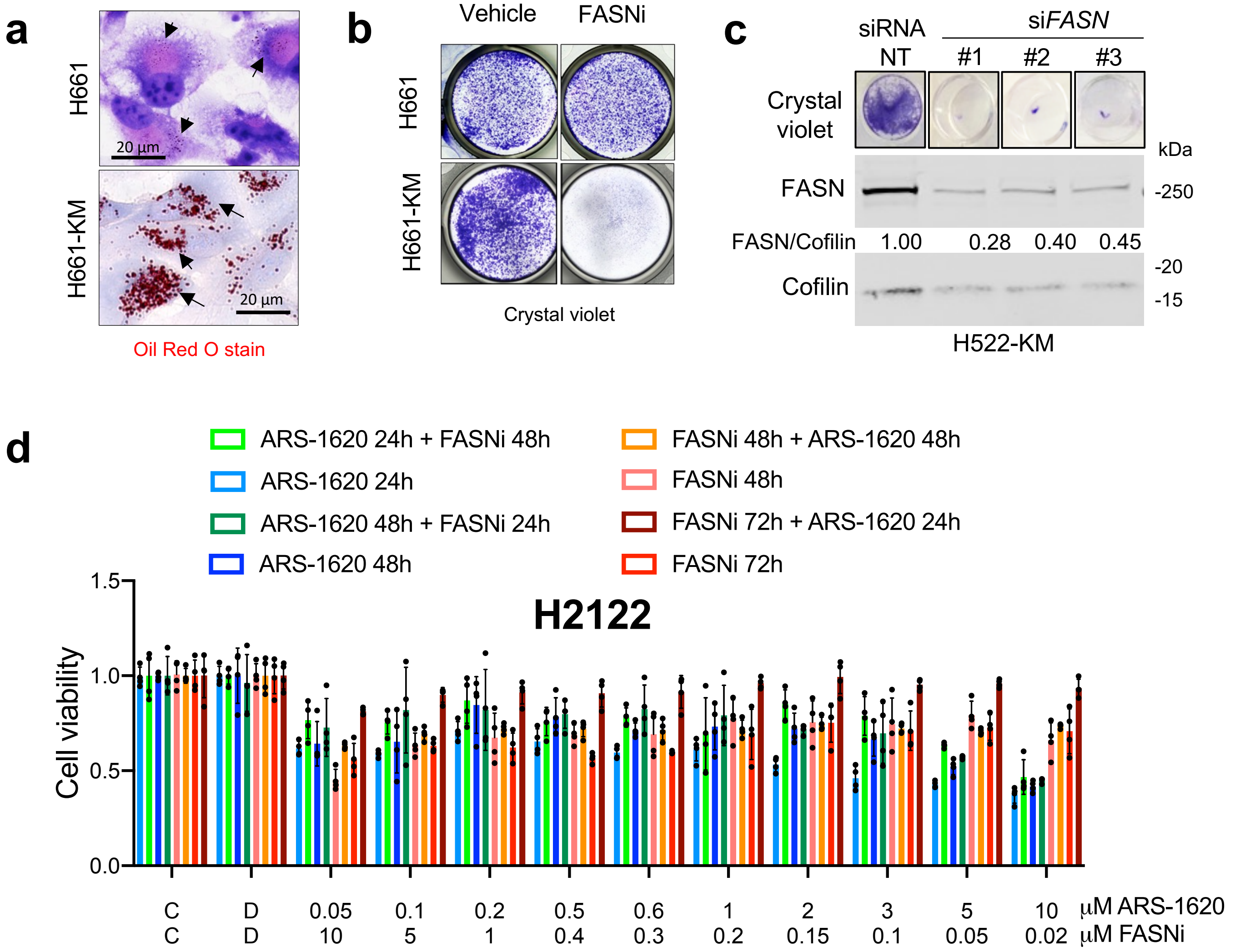

Supplementary Figure 4. KM is sufficient to induce dependency on FASN. (a) Oil red O staining for lipid droplets in the H661 (KRAS-WT) parental cell line and H661 transduced with KM (n=2 biological independent experiments). **(b)** Crystal violet assay of the indicated cell lines treated with vehicle or FASNi (0.2 μM). **(c)** Crystal violet assay of H522-KM cells transfected with the indicated siRNAs and correspondent immunoblot of FASN. NT, not targeting. **(d)** MTT cell viability assay in KM-G12C cell line H2122 treated as indicated (n=4 biologically independent samples). Data are represented as mean ± SD.

Supplementary Figure 5

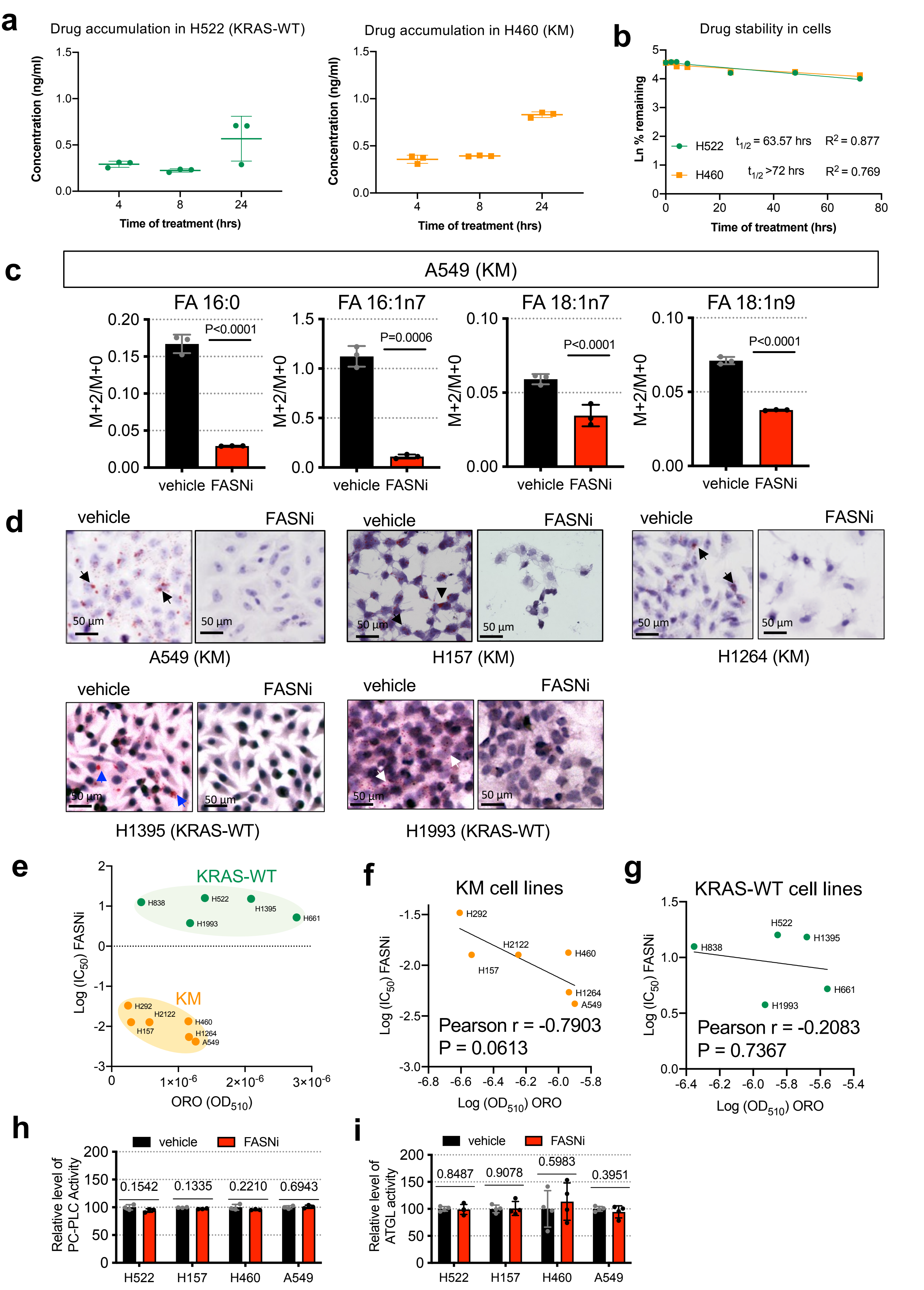

Supplementary Figure 5. **FASNi uptake, stability and activity in KM and KRAS-WT LC.**

(a, b) Intracellular accumulation and stability of FASNi in H522 (KRAS-WT) and H460 (KM) cell lines (n=3 replicates) assessed by LC/MS. $t_{1/2}$= drug half-life. **(c)** Quantification of newly synthesized FA via GC/MS in A549 cells treated as indicated. M+2/M+0 ratio is reported. Palmitate, FA 16:0; palmitoleate, FA 16:1n7; stearate, FA 18:0; vaccenate, FA 18:1n7; oleate, FA 18:1n9 (n=3 independent experiments). Data are reported mean ± SD. (**d**) Oil red O staining for detection of lipid droplets (arrows) in the indicated cell lines (n=2 independent experiments). **(e)** Principal component analysis plot performed using Oil Red O (ORO) OD510 and correspondent Log(IC_{50}) FASNi of the indicated cell lines. (**f**) Pearson correlation between ORO (OD_{510}) and correspondent Log(IC_{50}) FASNi in KM and (**g**) KRAS-WT LC cell lines. P values >0.05, not significant. (**h, i**) Relative activity of PC-specific phospholipase C (PC-PLC) and of adipose triglyceride lipase (ATGL) in the indicated LC cell lines (n=3 and n=4 biologically independent samples). Data are expressed as mean ± SD (a, b-d, g, h). Statistical significance was assessed using unpaired two-tailed Student's t test.

Supplementary Figure 6

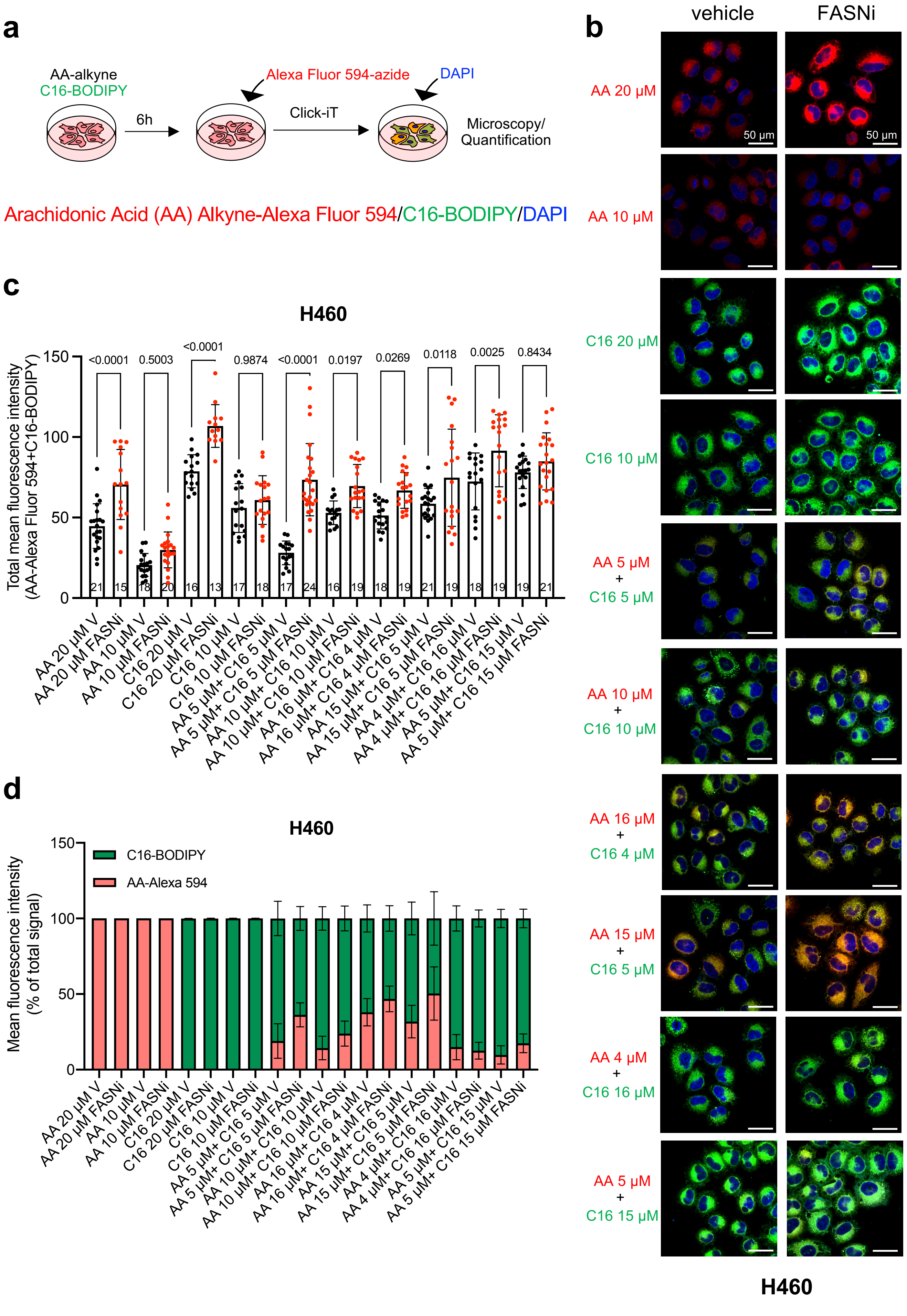

Supplementary Figure 6. Extracellular availability of PUFA/SFA dictates their uptake by KMLC cells. (**a, b**) Schematic and representative images of the FA uptake assay. AA, Arachidonic acid. C16, palmitate. (**c**) Quantification of the total FA uptake by H460 KMLC cells under the indicated conditions. n in the bars represents number of cells analysed in 2 biologically independent samples. Data are expressed as mean ± SD (**d**) Relative quantification of AA (red) and C16 (green) uptake by H460 cells under the indicated conditions. Data are expressed as mean ± SD and represents % of total mean florescence intensity signal (BODIPY + Alexa 594). In (**c**) multiple t test.

Supplementary Figure 7

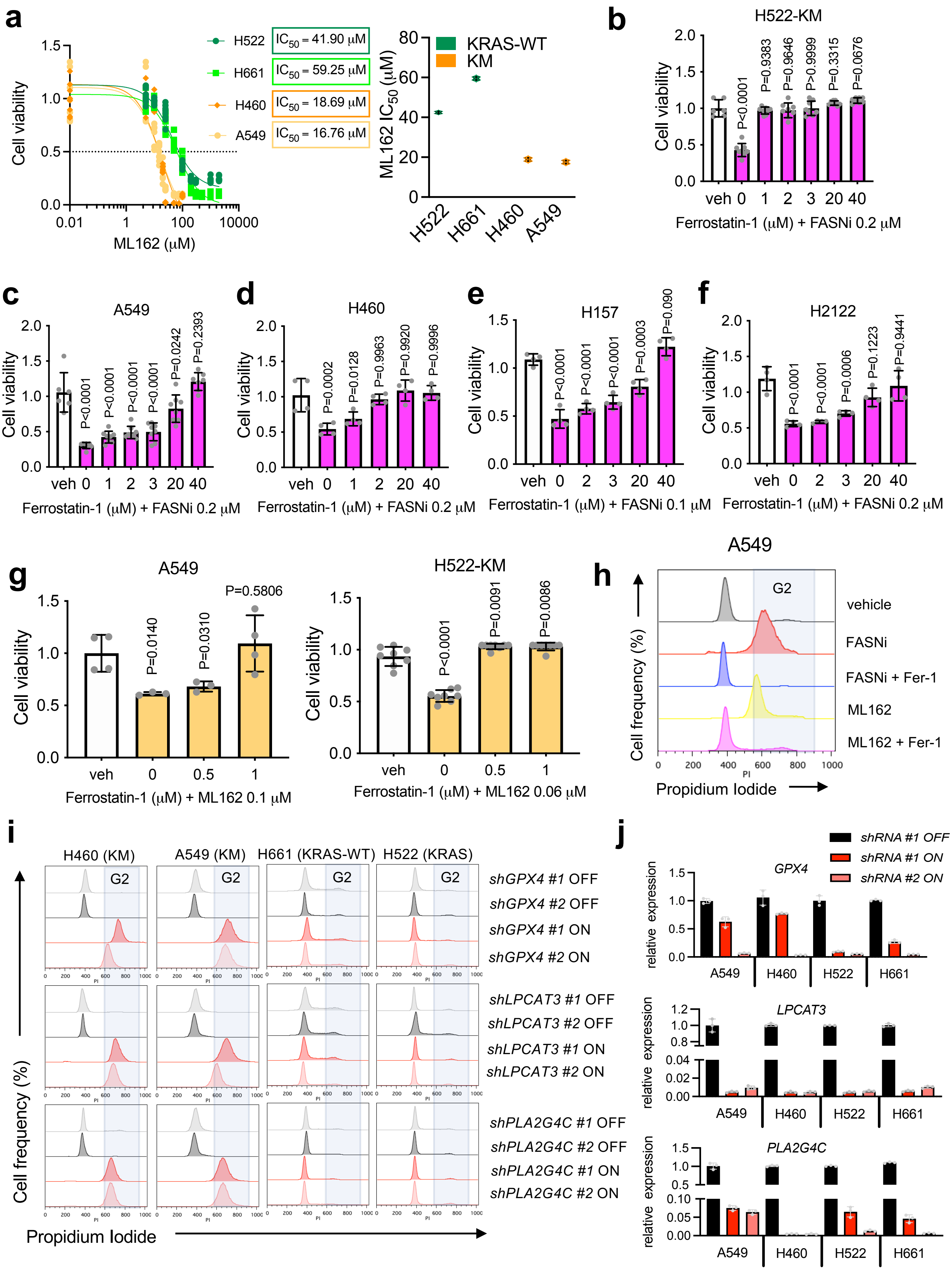

Supplementary Figure 7. KM expression lowers the threshold to ferroptosis induction. (a) MTT viability assay of the indicated KM and KRAS-WT LC cells treated with increasing concentrations of ML162 (GPX4 inhibitor). IC_{50} values are represented in the bar graph (n=2 independent experiments). **(b-f)** MTT viability assay of the indicated cell lines treated with FASNi or (**g**) ML162 in combination with increasing concentration of the ferroptosis inhibitor Ferrostatin-1. **(h, i)** Cell cycle analysis of the indicated cell lines treated as reported or transfected with the indicated inducible shRNAs. OFF/ON = -/+ doxy. Cell populations indicate singlets in the FL2-W/FL2-A gate. Refer to Supplementary Figure 11 for a representative gating strategy **(j)** RNA expression of *GPX4, LPCAT3* and *PLA2G4C* before or after induction of the shRNAs (n=3 biologically independent samples). Bars represent mean ± SD. In (c, d) unpaired two-tailed student t test.

Supplementary Figure 8

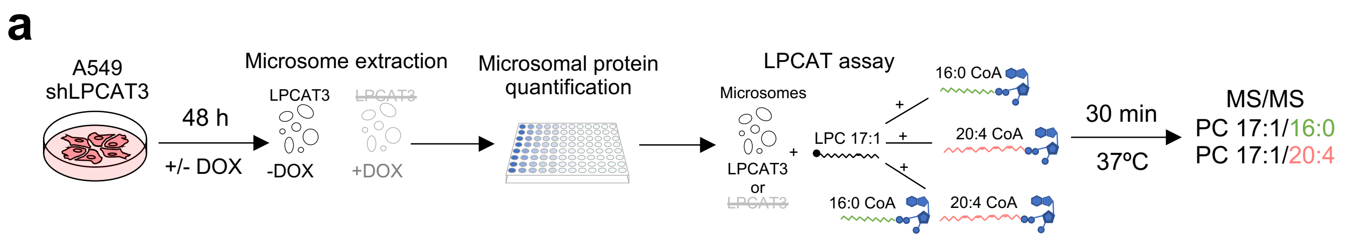

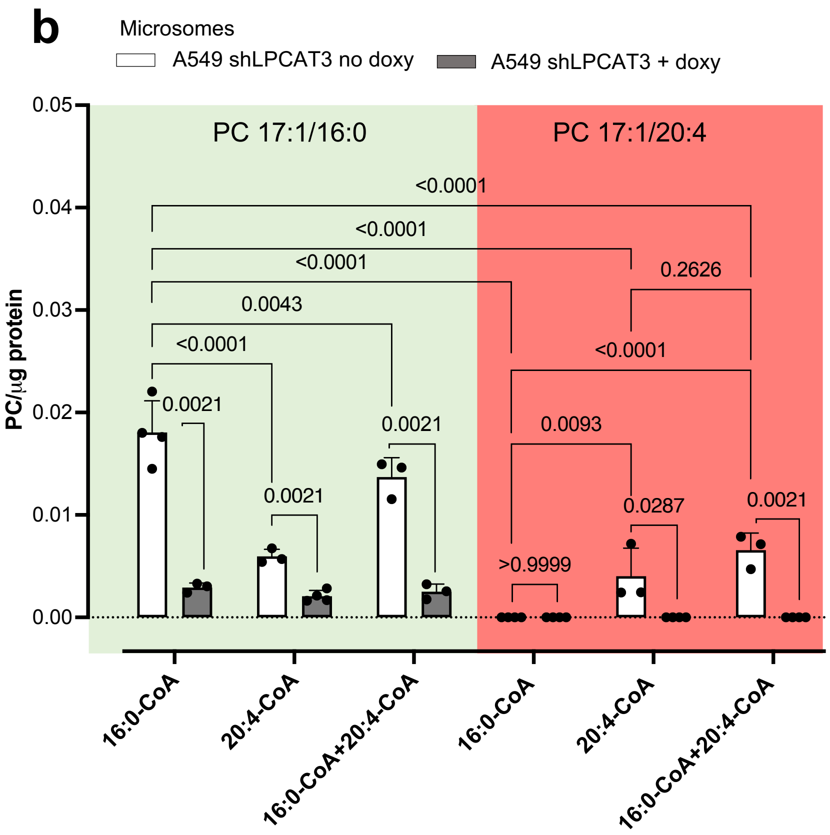

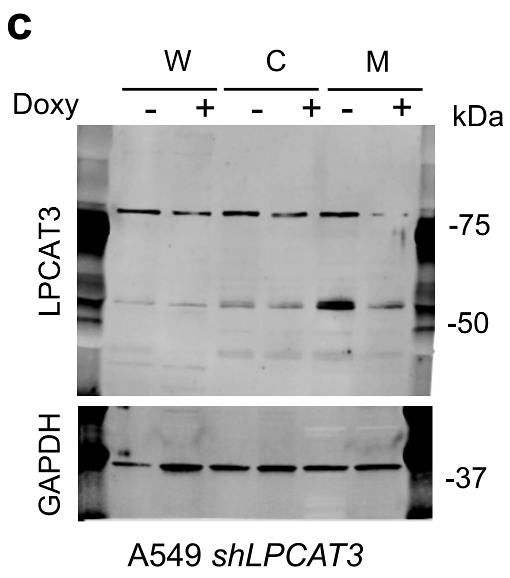

Supplementary Figure 8. LPCAT3 substrate choice is determined by PUFA and SFA availability in KMLC. (a) Schematic representation of the experimental approach. (**b**) HPLC-MS/MS quantification of PC 17:1/16:0 and PC 17:1/20:4 deriving from re-acylation of exogenously provided LysoPC 17:1 with either 16:0-CoA or 20:4 CoA, in presence of microsomes +/- doxy (*i.e.* +/- *shLPCAT3*). Data are expressed as mean ± SD (n=3 biologically independent samples). **(c)** Immunoblot for LPCAT3 and GAPDH on whole cell (W), cytoplasmic (C) and microsomal (M) lysates of A549 shLPCAT3 +/- doxy (n=2 independent experiments). In (b) multiple unpaired two-tailed t test followed by Benjamini, Krieger and Yekutieli FDR.

Supplementary Figure 9

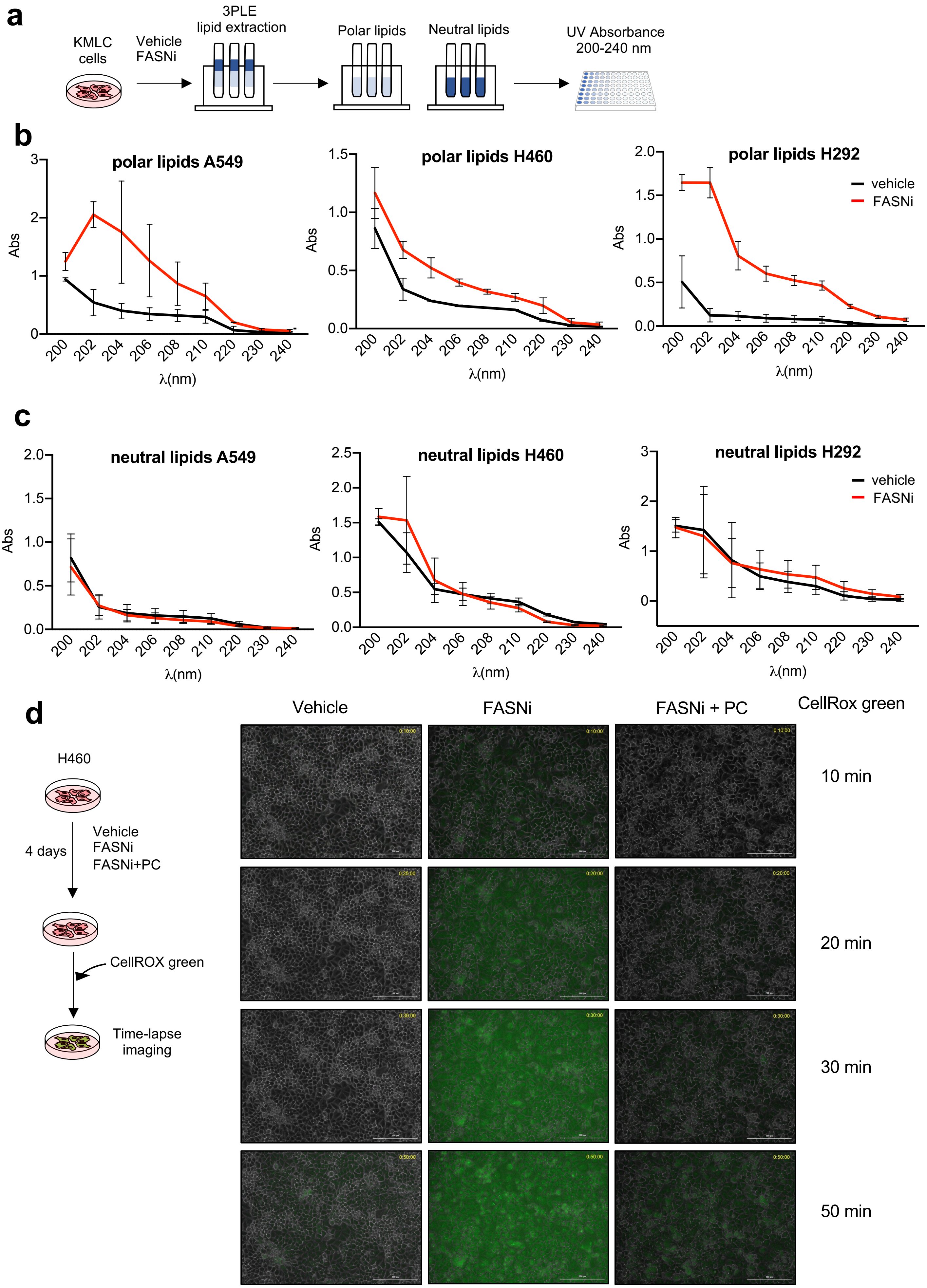

Supplementary Figure 9. FASNi causes phospholipid peroxidation. (a) Schematic workflow and (**b, c)** quantification of lipid peroxidation by UV absorbance in the polar and neutral lipid fractions of the indicated KMLC cell lines (for A549 n=2, for H460 and H292 n=3 biologically independent experiments. Data are expressed as mean ± SD. **(d)** Representative time lapse snapshots of ROS production in H460 treated as indicated. CellROX green accumulation indicates oxidative stress. See also Supplementary videos 1-3.

Supplementary Figure 10

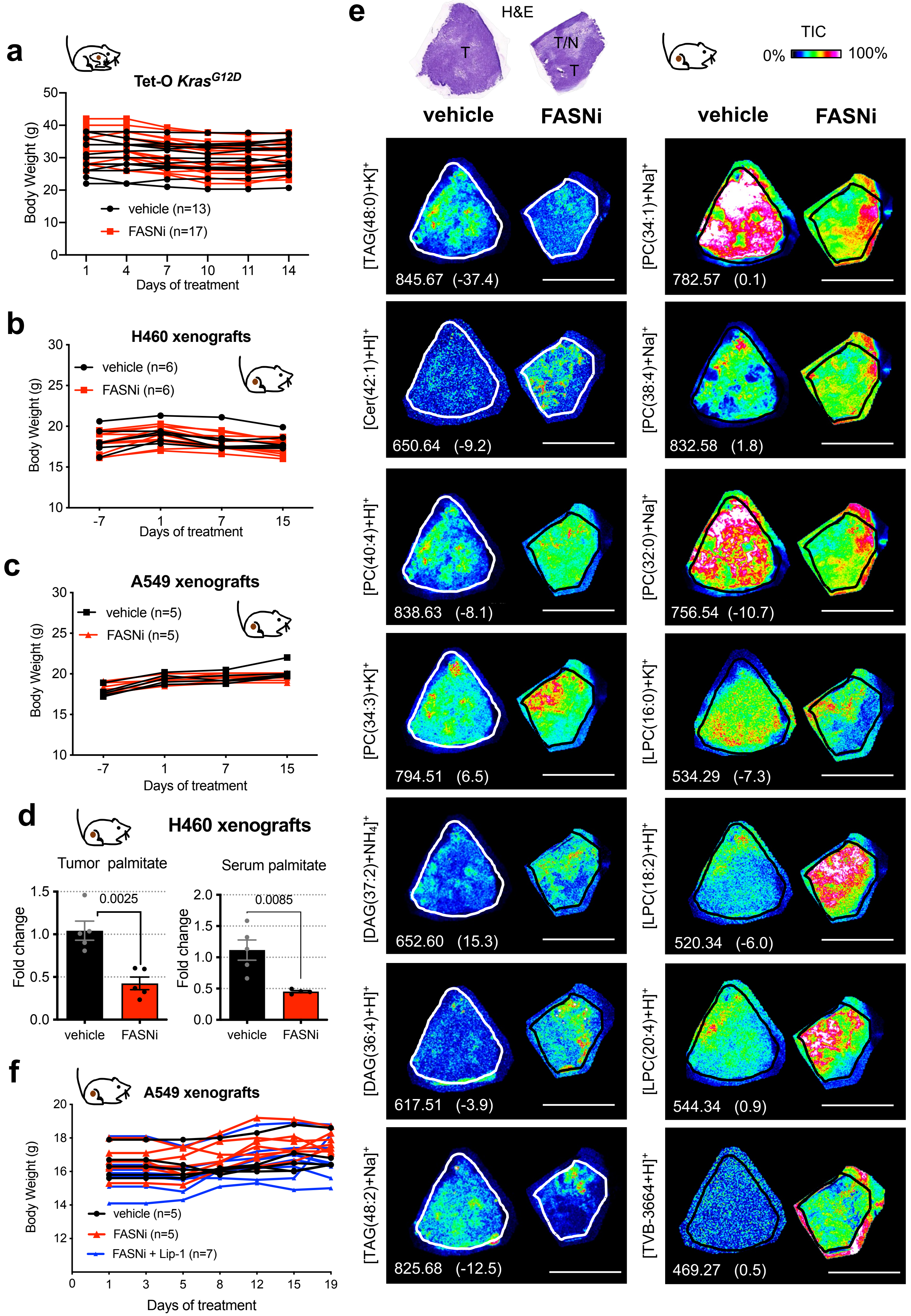

Supplementary Figure 10. Body weight, palmitate quantification and MALDI imaging of KMLC mouse models. (a-c) Body weight of TetO-*Kras* and xenograft-bearing mice treated as indicated. **(d)** Palmitate quantification in xenografts and sera of NOD/SCID mice (n=5 mice/group). Data are expressed as mean ± SD**.** Statistics indicates unpaired two-tailed t test. **(e)** Representative MALDI images of the indicated lipid species in A549 xenografts. Rainbow scale represents % ion intensity normalized against the total ion count (TIC). Corresponding H&E and histological annotation are shown. T, tumor; N, necrotic area. Observed m/z and mass error (ppm) values are indicated for each lipid species. Refer to Supplementary Data 1 and 8 for the complete tentative MALDI lipid annotation and relative quantification (**f)** Body weight of A549 xenograft-bearing mice treated as indicated. Lip-1, liproxstatin-1.

Supplementary Figure 11

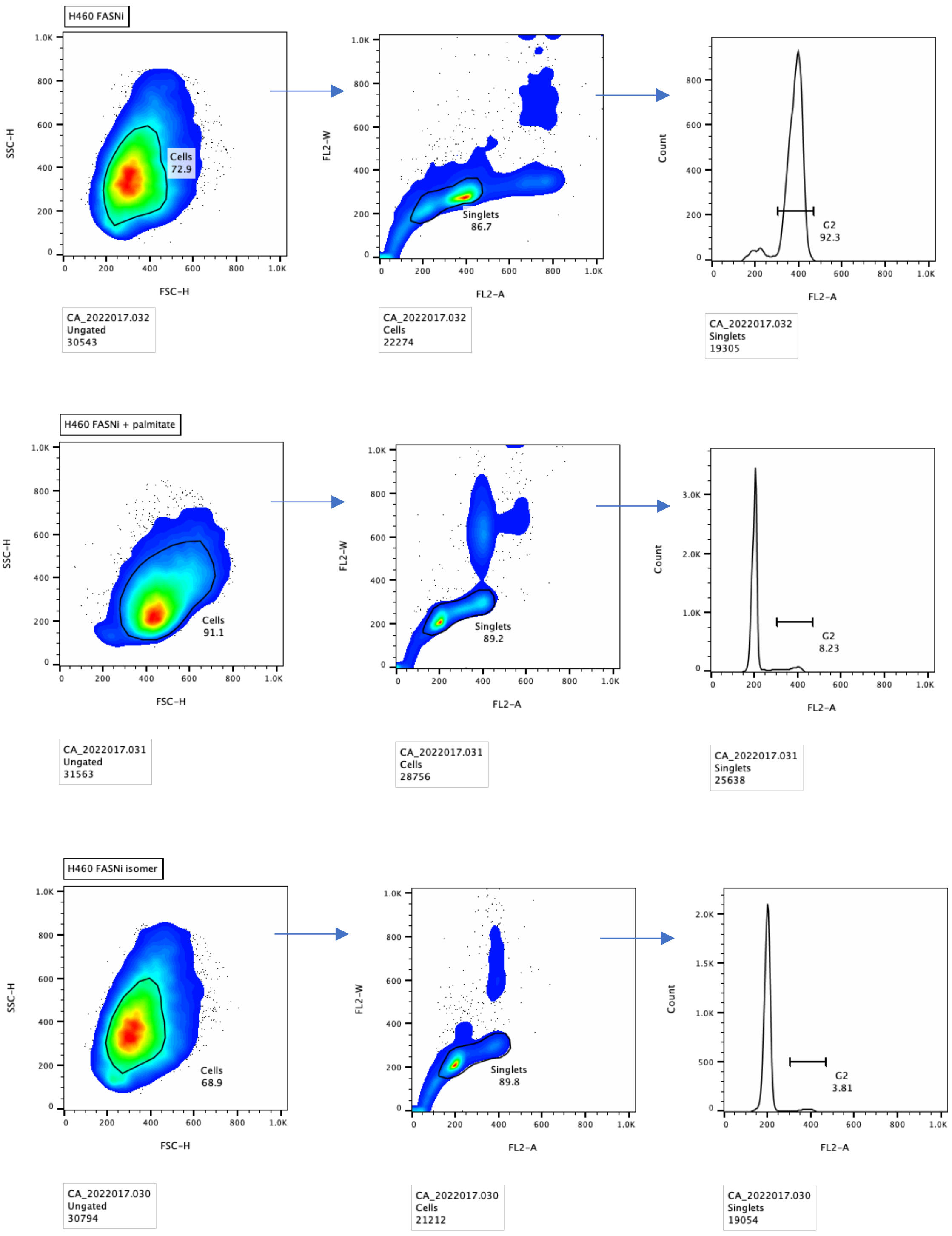

Supplementary Figure 11. Gating strategy for cell cycle facs experiments. Cell population is gated in SSC-H/FSC-H to remove debris (gate “Cells”). “Cells” population is then gated in FL2-W/FL2-A to select only single cells (gate “Singlets”). The “Singlets” population is represented as histogram over FL2-A. G2 peak is gated in the “Singlets”.
