# Supplementary material for: Targeting de novo lipogenesis and the Lands cycle induces ferroptosis in KRAS-mutant lung cancer
Source: Nat Commun. 2022 Jul 26;13:4327. doi: 10.1038/s41467-022-31963-4 (PMC9325712; doi:10.1038/s41467-022-31963-4)
Supplement: Supplementary file 3 — Description of Additional Supplementary Files [file 41467_2022_31963_MOESM3_ESM.pdf]

Description of Additional Supplementary Files

File Name: Supplementary Movie 1
Description: CellRox green live imaging in H460 cells treated with vehicle for 72 hours.

File Name: Supplementary Movie 2
Description: CellRox live green imaging in H460 cells treated with TVB-3664 (0.2 μM) for 72 hours.

File Name: Supplementary Movie 3
Description: CellRox live green imaging in H460 cells treated with TVB-3664 (0.2 μM) and 16:0-18:1 PC (50 μM) for 72 hours.

File Name: Supplementary Data 1
Description: Tentative lipid assignment of MALDI data.

File Name: Supplementary Data 2
Description: MALDI annotation and relative quantification in TetO-KM mice

File Name: Supplementary Data 3
Description: MALDI annotation and relative quantification in LC PDXs.

File Name: Supplementary Data 4
Description: MALDI annotation and relative quantification in primary human LC specimens.

File Name: Supplementary Data 5
Description: Clinical annotation, genotype and FASN immunoreactive score quantification of YTMA310 and TMA4.

File Name: Supplementary Data 6
Description: Differentially expressed genes identified via RNA-seq.

File Name: Supplementary Data 7
Description: Mutational status of the lung cancer cell lines used.

File Name: Supplementary Data 8
Description: MALDI annotation and relative quantification in A549 mouse xenografts.

File Name: Supplementary Data 9
Description: siRNAs, primer sequences and statistics of the siRNA screening in fig.5h,g.
