# Supplementary figures and images for: Targeting de novo lipogenesis and the Lands cycle induces ferroptosis in KRAS-mutant lung cancer
Source: Nat Commun. 2022 Jul 26;13:4327. doi: 10.1038/s41467-022-31963-4 (PMC9325712; doi:10.1038/s41467-022-31963-4)

## Slide 1
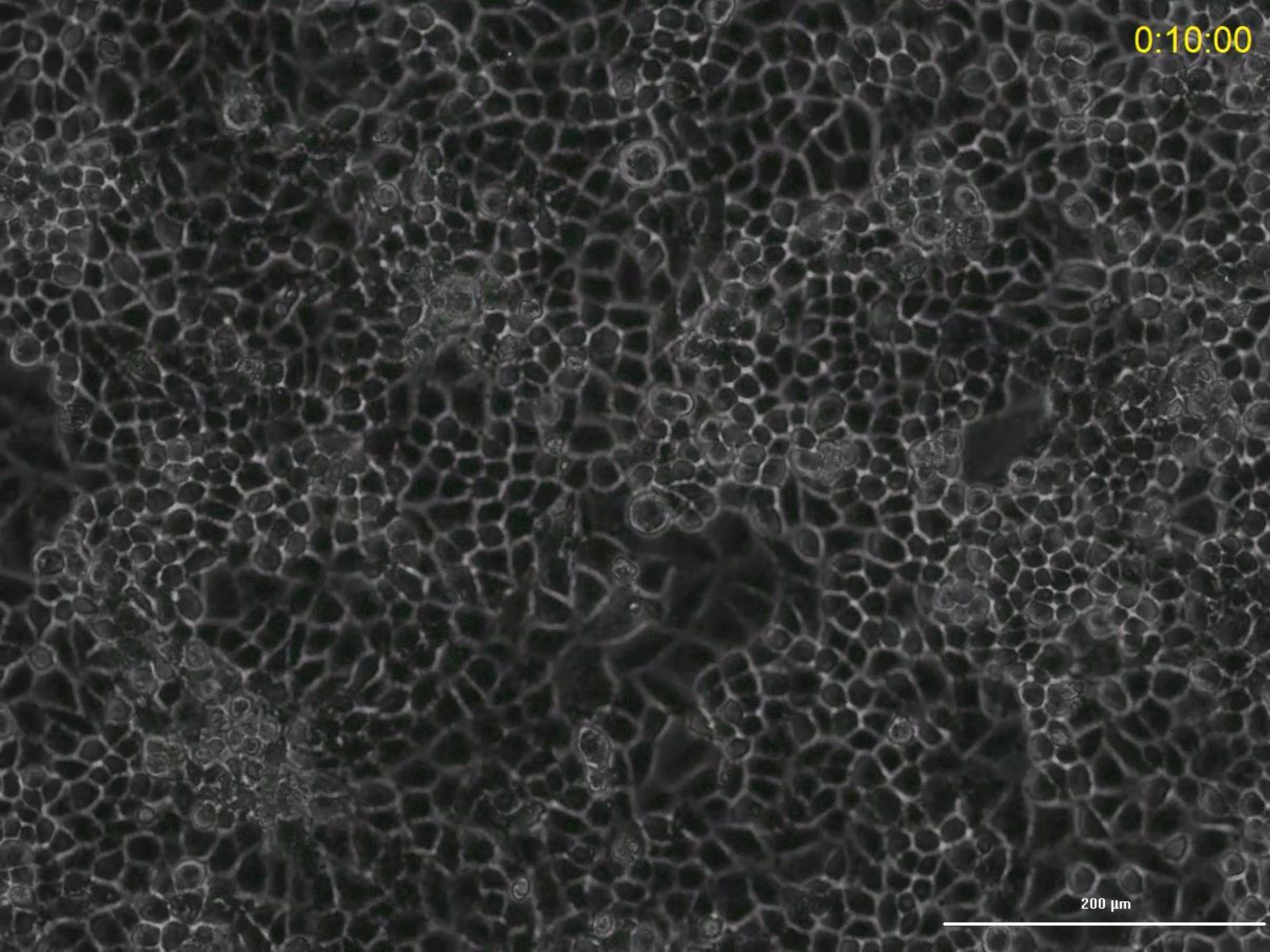

Supplement: Supplementary file 4 — Supplementary Movie 1 [file 41467_2022_31963_MOESM4_ESM.pptx]

## Slide 1
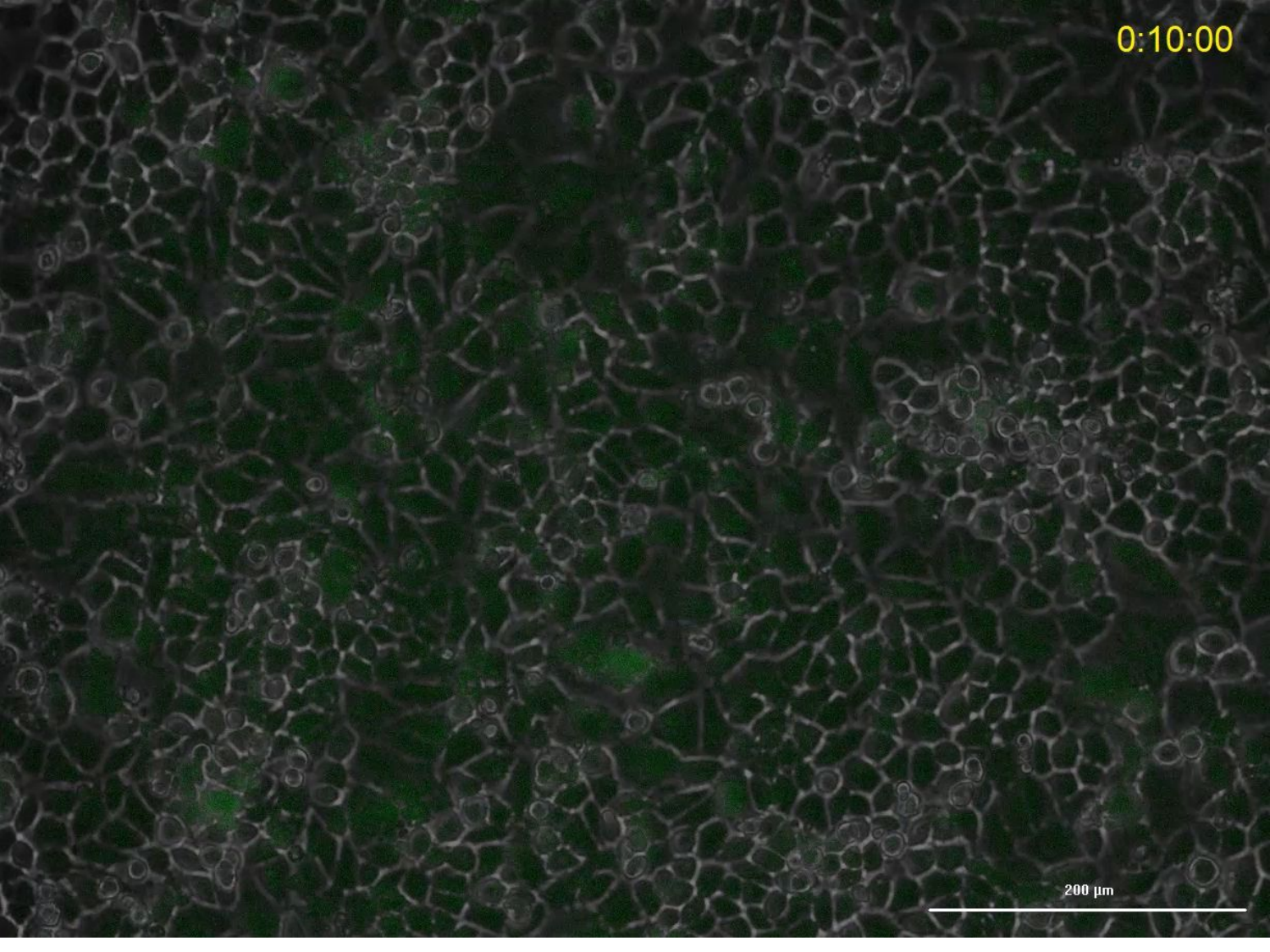

Supplement: Supplementary file 5 — Supplementary Movie 2 [file 41467_2022_31963_MOESM5_ESM.pptx]

## Slide 1
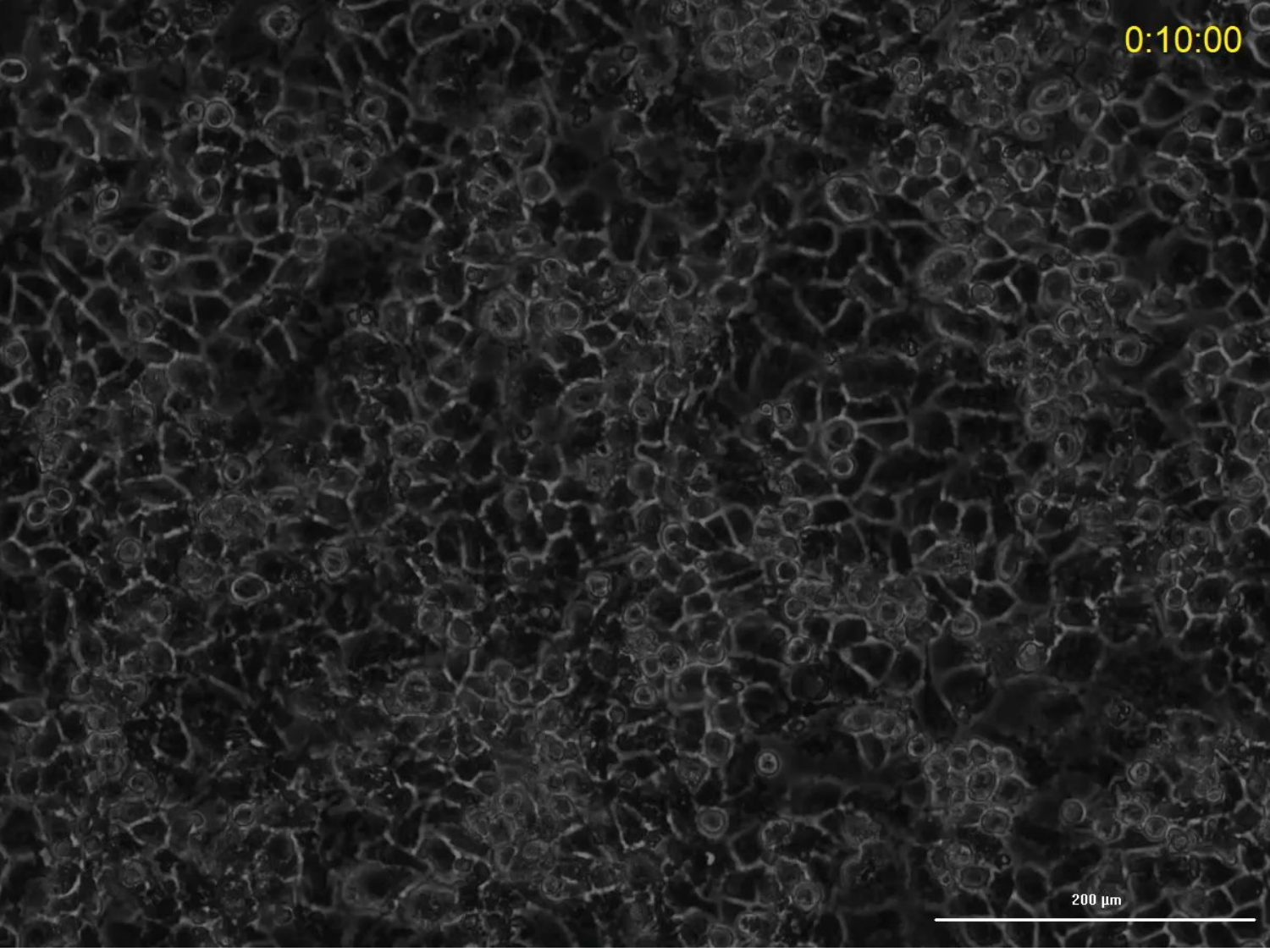

Supplement: Supplementary file 6 — Supplementary Movie 3 [file 41467_2022_31963_MOESM6_ESM.pptx]
