# Supplementary material for: Targeting de novo lipogenesis and the Lands cycle induces ferroptosis in KRAS-mutant lung cancer
Source: Nat Commun. 2022 Jul 26;13:4327. doi: 10.1038/s41467-022-31963-4 (PMC9325712; doi:10.1038/s41467-022-31963-4)
Supplement: Supplementary file 16 — Reporting Summary [file 41467_2022_31963_MOESM16_ESM.pdf]

nature portfolio

Corresponding author(s): Pier Paolo Scaglioni

Last updated by author(s): Apr 1, 2022

Reporting Summary

Nature Portfolio wishes to improve the reproducibility of the work that we publish. This form provides structure for consistency and transparency in reporting. For further information on Nature Portfolio policies, see our Editorial Policies and the Editorial Policy Checklist.

Statistics

For all statistical analyses, confirm that the following items are present in the figure legend, table legend, main text, or Methods section.

n/a	Confirmed	
☐	☒	The exact sample size (*n*) for each experimental group/condition, given as a discrete number and unit of measurement
☐	☒	A statement on whether measurements were taken from distinct samples or whether the same sample was measured repeatedly
☐	☒	The statistical test(s) used AND whether they are one- or two-sided<br>*Only common tests should be described solely by name; describe more complex techniques in the Methods section.*
☒	☐	A description of all covariates tested
☐	☒	A description of any assumptions or corrections, such as tests of normality and adjustment for multiple comparisons
☐	☒	A full description of the statistical parameters including central tendency (e.g. means) or other basic estimates (e.g. regression coefficient) AND variation (e.g. standard deviation) or associated estimates of uncertainty (e.g. confidence intervals)
☐	☒	For null hypothesis testing, the test statistic (e.g. F, t, r) with confidence intervals, effect sizes, degrees of freedom and P value noted<br>*Give P values as exact values whenever suitable.*
☒	☐	For Bayesian analysis, information on the choice of priors and Markov chain Monte Carlo settings
☒	☐	For hierarchical and complex designs, identification of the appropriate level for tests and full reporting of outcomes
☐	☒	Estimates of effect sizes (e.g. Cohen's *d*, Pearson's *r*), indicating how they were calculated

Our web collection on statistics for biologists contains articles on many of the points above.

Software and code

Policy information about availability of computer code

Data collection	HD imaging version 1.4; Masslynx 4.1; Analyst TF version 1.7.1; Agilent MassHunter data acquisition software for 5975/5977; POLARstar Omega version 1.20; NextSeq System Suite version 2.2.0; Gen5 version 3.06: ZEN 2010 B SP1 (Zeiss);BD FACSDiva version 8.0.1
Data analysis	msIQuant version 2.0 ; ImageJ version 1.46; LipPy (in-house script); Microsoft Excel for Mac version 16.34; GraphPad Prism version 9; STAR (v3.7.3a); Gencode annotation for human (version v37); featureCounts (v2.0.1); DESeq2 version 1.34; sva version 3.42; Enrichr (https://amp.pharm.mssm.edu/Enrichr/#); msIQuant version 2.0; LipidSearch 4.1.

For manuscripts utilizing custom algorithms or software that are central to the research but not yet described in published literature, software must be made available to editors and reviewers. We strongly encourage code deposition in a community repository (e.g. GitHub). See the Nature Portfolio guidelines for submitting code & software for further information.

Data

Policy information about availability of data

All manuscripts must include a data availability statement. This statement should provide the following information, where applicable:

- Accession codes, unique identifiers, or web links for publicly available datasets
- A description of any restrictions on data availability
- For clinical datasets or third party data, please ensure that the statement adheres to our policy

RNA-seq data were deposited in GEO under the accession number GSE168782 https://www.ncbi.nlm.nih.gov/geo/query/acc.cgi?acc=GSE168782. MALDI-MS data have been submitted to the DRYAD database uder DOI https://doi.org/10.5061/dryad.ght76hq1. The publicly available databases LIPID MAPS (https://www.lipidmaps.org/) , METLIN (https://www.metlin.scripps.edu) and Human Metabolite Database (https://www.hmdb.ca) were used for the lipidomic annotation. The Cancer Cell Line Encyclopedia database (CCLE, https://sites.broadinstitute.org/ccle/datasets) was used for FASN expression data.

Source data are provided with the paper. All data supporting the findings of this work are available within this article, Supplementary Information, Source data and the peer review file. Further requests for resources, reagents and data should be directed to and will be fulfilled by the corresponding author.

Field-specific reporting

Please select the one below that is the best fit for your research. If you are not sure, read the appropriate sections before making your selection.

☒ Life sciences ☐ Behavioural & social sciences ☐ Ecological, evolutionary & environmental sciences

For a reference copy of the document with all sections, see nature.com/documents/nr-reporting-summary-flat.pdf

Life sciences study design

All studies must disclose on these points even when the disclosure is negative.

Sample size	The minimum number of samples/animals to obtain a power of at least 95% with alpha equal to 0.05 was calculated using clincalc.com based on previous experiments. Sample size equal to or greater than 3 for all the analyses
Data exclusions	Outliers were excluded using GraphPad Prism function ROUT with Q=1%.
Replication	All experiments were repeated at least twice. All attempts of replication were successful
Randomization	Animals were randomly assigned to control and treatment groups. MS/MS replicates were injected in a random order. For all the other experiments allocation of cell lines was done according to the genotype and treatment to allow correct interpretation of the data.
Blinding	Samples for metabolic analyses were always de-identified. siRNA screening and TMA analysis were performed blindly. For all the other experiments investigators were not blinded in order to allow the correct analysis and interpretation of the data.

Reporting for specific materials, systems and methods

We require information from authors about some types of materials, experimental systems and methods used in many studies. Here, indicate whether each material, system or method listed is relevant to your study. If you are not sure if a list item applies to your research, read the appropriate section before selecting a response.

Materials & experimental systems

n/a	Involved in the study	
☐	☒	Antibodies
☐	☒	Eukaryotic cell lines
☒	☐	Palaeontology and archaeology
☐	☒	Animals and other organisms
☐	☒	Human research participants
☒	☐	Clinical data
☒	☐	Dual use research of concern

Methods

n/a	Involved in the study	
☒	☐	ChIP-seq
☐	☒	Flow cytometry
☒	☐	MRI-based neuroimaging

Antibodies

Antibodies used	FASN (C20G5) rabbit mAb (CST #3180, 1:1000 dil); SCD1 (C12H5) rabbit mAb (CST #2794, 1:1000 dil); AMPKα Antibody rabbit pAb (CST #2532, 1:1000 dil); phospho-AMPKα (Thr172) (40H9) rabbit mAb (CST #2535, 1:1000 dil); ACC1 (C83B10) Rabbit mAb (CST #3676, 1:1000 dil); Phospho-ACC1 (Ser79) (D7D11) rabbit mAb (CST #11818, 1:1000 dil); KRAS (234-4.2) mouse mAb (Millipore Sigma #MABS194, 1:700 dil); pan RAS (C-4) mAb (SCBT #SC-166691, 1:500 dil); GAPDH (6C5) mAb (SCBT #SC-32233, 1:500 dil); LPCAT3 rabbit polyclonal (Novus Bio #NBP3-04752, 1:1000 dil); Cofilin (D3F9) rabbit mAb (CST #5175, 1:1000 dil); Donkey anti-rabbit IgG (H+L) crossed-absorbed secondary antibody-DyLight 680 (invitrogen #SA5-10042, dil 1:5000); Donkey anti-mouse IgG (H+L) crossed-absorbed secondary antibody-DyLight 680 (invitrogen #SA5-10170, dil 1:5000); Goat anti-rabbit IgG (H+L) crossed-absorbed secondary antibody-DyLight 800 (invitrogen #SA5-10036, dil 1:5000); Goat anti-mouse IgG (H+L) crossed-absorbed secondary antibody-DyLight 800 (invitrogen #SA5-10176, dil 1:5000).
Validation	FASN (SCBT, SC-55580) was validated by Human Protein Atlas for IHC applications and in our lab in preliminary IHC staining.<br>All the other antibodies were validated by the manufacturer's for western blot against Human and Mouse proteins.

Eukaryotic cell lines

Policy information about cell lines

Cell line source(s)	Human NSCLC cell lines were obtained from the Hamon Cancer Center cell line repository (UT Southwestern Medical Center).

Cell line source(s)	Dr. Monte M. WislCCL-186ow kindly provided mouse KMLC cell lines 238N1, 802T4, 368T1, 593T5 derived from LSL-KrasG12D lung tumours. IMR-90 were from ATCC (# CCL-186)
Authentication	Cell lines were authenticated by DNA fingerprints for cell-line individualization using Promega Stem Elite ID system, a short tandem repeat (STR)-based assay, at UT Southwestern Medical Center Genomics Core.
Mycoplasma contamination	All cell lines were mycoplasma free (e-Myco Kit; Boca Scientific)
Commonly misidentified lines (See ICLAC register)	no commonly misidentified cell lines were used in the study

Animals and other organisms

Policy information about studies involving animals; ARRIVE guidelines recommended for reporting animal research

Laboratory animals	All animal studies involved Mus musculus species: 6 week old NOD/SCID females; 8 week old CCSP-rtTA/Tet-op-Kras (FVB/SV129 mixed background) females and males
Wild animals	The study did not involve wild animals
Field-collected samples	The study did not involve field-collected samples
Ethics oversight	All animal studies were approved by the Institutional Animal Care and Use Committee (IACUC) at University of Cincinnati (protocols 18-04-16-01 and 20-11-30-02 18-04-16-01).

Note that full information on the approval of the study protocol must also be provided in the manuscript.

Human research participants

Policy information about studies involving human research participants

Population characteristics	The tumor samples collected and stored in our tumor bank are obtained from consecutive patients that presented to UC Medical Center and Hamon Cancer Center at UT Southwestern Medical Center for treatment.
Recruitment	A possible bias could be introduced by the fact that patients that are health conscious accept to participate. All patients were consented and agreed to participate to the study
Ethics oversight	All human studies were approved by the Institutional Review Board (IRB) at University of Cincinnati and UT Southwestern Medical Center (protocols 2013-4722 and 2013-4520)

Note that full information on the approval of the study protocol must also be provided in the manuscript.

Flow Cytometry

Plots

Confirm that:

☒ The axis labels state the marker and fluorochrome used (e.g. CD4-FITC).

☒ The axis scales are clearly visible. Include numbers along axes only for bottom left plot of group (a 'group' is an analysis of identical markers).

☒ All plots are contour plots with outliers or pseudocolor plots.

☒ A numerical value for number of cells or percentage (with statistics) is provided.

Methodology

Sample preparation	We performed cell cycle analysis after 4 days of pharmacological treatments, or 72 hr after siRNA transfection or doxy-dependent induction of shRNAs, respectively. The cells were fixed in ice-cold 70% ethanol for 30 min. RNA digestion was performed with 100 g/mL RNase A (Sigma Aldrich, R6513) for 15 min at 37°C. DNA staining was performed with 50 µg/mL propidium iodide (Sigma Aldrich, P4170) for 30 min at 37°C
Instrument	BD LSRFortessa™
Software	Data were collected with BD FACSDiva version 8.0.1 and analyzed with FlowJo version 8.7
Cell population abundance	The acquisitions were performed with at least 30000 events per sample.
Gating strategy	SSC-A /FSC-A gate was used to exclude debris (<200 SSC-A/FSC-A), then singlets were gated using PI-A/PI-W and visualized as PI-A histograms. Y axis represents the frequency with respect to total singlets.

☒ Tick this box to confirm that a figure exemplifying the gating strategy is provided in the Supplementary Information.
